# Supplementary material for: Orphan cytochrome P450 20a1 CRISPR/Cas9 mutants and neurobehavioral phenotypes in zebrafish
Source: Sci Rep. 2021 Dec 13;11:23892. doi: 10.1038/s41598-021-03068-3 (PMC8669017; doi:10.1038/s41598-021-03068-3)
Supplement: Supplementary file 1 — Supplementary Information 1. [file 41598_2021_3068_MOESM1_ESM.pdf]

Supplementary Information

Orphan cytochrome P450 20A1 CRISPR/Cas9 mutants and neurobehavioral phenotypes in zebrafish

Nadja R. Brun, Matthew C. Salanga, Francisco X. Mora-Zamorano, David C. Lamb

Jared V. Goldstone, and John J. Stegeman

Table S1. Primer sequences for endpoint PCR and CRISPR-Cas sgRNA target and synthesis sequences.

Sequence Name	Barcode ID	Primer sequence (5’)	Cycling condition	Polymerase	Product length (bp)
cyp20-wtP2-F	1025475438	TGTCATCATTCTGATTGGTGCC	98˚ - 3 min - (1x); 98˚ - 10 sec, 65˚ - 10 sec, 72˚ - 20 sec (35x); 72˚ - 5 min (1x)	NEB Q5	437
cyp20-wtP2-R	1025479906	GTTGATGTGTTGTCGTAGTTGG			
cyp20-ms61P1-F	1025475440	TAGACGAGCTTCTGGTGTTACC	98˚ - 3 min - (1x); 98˚ - 10 sec, 65˚ - 10 sec, 72˚ - 20 sec (35x); 72˚ - 5 min (1x)	NEB Q5	277
cyp20-ms61P1-R	1025475441	CAGTTAGGGTTGATGTGTTGTCG			
cyp20_60_wtF	1033095856	GACGAGCTTCTGGTGTACCTGG	98˚ - 3 min - (1x); 98˚ - 10 sec, 68˚ - 10 sec, 72˚ - 20 sec (35x); 72˚ - 5 min (1x)	NEB Q5	304
cyp20_60_wtR	1033095857	CACTGCGGCTACTCACTGGT			
cyp20_60_mutF	1033095860	TTACGCCCCTGTCTTGCAGT	98˚ - 3 min - (1x); 98˚ - 10 sec, 65˚ - 10 sec, 72˚ - 30 sec (35x); 72˚ - 5 min (1x)	NEB Q5	744
cyp20_60_mutR	1033095861	CTGTTGGGTTTAGTCCAGGTTAAAAG			
dr.cyp20a1-F	1023898079	ACCATGCTAGATTTTGCCATTTTTGCTGTG			
dr.cyp20a1-R	1023898080	TCAGTTTCTCTTGCTGACCGTG			
z.cyp20a1-5'utr-F	1023898081	GTAGTCGAGTACCGATCTAGAGG			
z.cyp20a1-3'utr-R	1023898082	GTGTAATTCCCATCCTCCAGAGG			
dr.cyp20a1.T7.sg1	1023342952	GATTAATACGACTCACTATAGGACGAGCTTCTGGTGTACCGTTTTAGAGCTAGAAATAGC			
dr.cyp20a1.T7.sg2	1023342953	GATTAATACGACTCACTATAGGTTGATGTGTTGTCGTAGTGTTTTAGAGCTAGAAATAGC			
sgRNA Universal reverse primer		AAAAGCACCGACTCGGTGCCACTTTTTCAAGTTGATAACGGACTAGCCTTATTTTAACTTGCTATTTCTAGCTCTAAAAC			

dr.cyp20a1.seqpF1	1023342954	ATCGCCAGCTCGTAGTTCAC
dr.cyp20a1.seqpR1	1023342955	CAGTCTTCAACTGTAAATGCAGC
dr.cyp20a1.seqpF2	1023342956	TCCTGATGGTCATTGTAGACG
dr.cyp20a1.seqpR2	1023342957	CAGGCGGACTGATAATTCAGG
CYP20A1_Dr_pENTRF	1018501550	CACCATGCTAGATTTTGCCA
CYP20A1_Dr_pENTRR	1018501551	TCTCTTGCTGACCGTGATCCA
zf_cyp20_f4	1017216446	TACAGGAGGTGGAAGGAAAGGTG
zf_cyp20_r4	1017216447	GACGACACCAAGGGCATAGATAAC

Table S2. Statistical results.

**Assay**	**Endpoint**	***cyp20a1-/-* Line**	**# of Trials**	**Total *n* (WT)**	**Total *n* (Cyp20A1)**	**Estimation Stats**	**Passed Normality Test**	**Unpaired t-test[1]**	**Mann-Whitney Test[2]**
Morphology	Swim Bladder Inflation	wh^{61}	3	18	18	Unpaired mean difference of *cyp20a1-/-* (n = 18) minus WT (*n* = 18) -30.6 [95CI -38.3; -22.8]	Some		U = 6, *p* < .001***
OMR	Right Grating (prior)	wh^{61}	3	107	111	Unpaired mean difference of *cyp20a1-/-* (*n* = 111) minus WT (*n* = 107) -0.555 [95CI -0.841; -0.283]	No		U = 4577, *p* = .003**
OMR	Right Grating (during)	wh^{61}	3	95	99	Unpaired mean difference of *cyp20a1-/-* (*n* = 99) minus WT (*n* = 95) 0.472 [95CI -0.0656; 0.973]	No		U = 4189, *p* = .190
OMR	Left Grating (prior)	wh^{61}	3	108	111	Unpaired mean difference of *cyp20a1-/-* (*n* = 111) minus WT (*n* = 108) -0.349 [95CI -0.668; -0.0377]	No		U = 5266, *p* = .121
OMR	Left Grating (during)	wh^{61}	3	100	102	Unpaired mean difference of *cyp20a1-/-* (*n* = 102) minus WT (*n* = 100) 0.267 [95CI -0.223; 0.753]	No		U = 4760, *p* = .414

OMR	Average Speed	wh^{61}	3	115	115	Unpaired mean difference of *cyp20a1-/-* (*n* = 115) minus WT (*n* = 115) -0.499 [95CI -0.712; -0.287]	Yes	t(228) = 4.711, *p* < .001***	
OMR	Maximum Speed	wh^{61}	3	115	115	Unpaired mean difference of *cyp20a1-/-* (*n* = 115) minus WT (*n* = 115) 1.11 [95CI -0.293; 3.13]	Some		U =5778, *p* = .098
OMR	Activity	wh^{61}	3	115	115	Unpaired mean difference of *cyp20a1-/-* (*n* = 115) minus WT (*n* = 115) -14.1 [95CI -21.3; -6.88]	No		U = 4492, *p* < .001***
OMR	Total Distance Traveled	wh^{61}	3	115	115	Unpaired mean difference of *cyp20a1-/-* (*n* = 115) minus WT (*n* = 115) -149 [95CI -213; -85.6]	Yes	t(228) = 4.706, *p* < .001***	
Ligth-Dark	Total Activity Dark	wh^{61}	3	71	65	Unpaired mean difference of *cyp20a1-/-* (*n* = 65) minus WT (*n* = 71) 566 [95CI 299; 824]	Yes	t(134) = 4.251, *p* < .0001****	
Ligth-Dark	Total Activity Light	wh^{61}	3	71	65	Unpaired mean difference of *cyp20a1-/-* (*n* = 65) minus WT (*n* = 71) 782 [95CI 448; 1130]	Yes	t(134) = 4.447, *p* < .0001****	
Ligth-Dark	Total Activity Dark	wh^{61}	5	120	120	Unpaired mean difference of *cyp20a1-/-* (*n* = 120) minus WT (*n* = 120) 659 [95CI 433; 883]	Some		U = 4200, *p* < .0001****
Ligth-Dark	Total Activity Light	wh^{61}	5	120	120	Unpaired mean difference of *cyp20a1-/-* (*n* = 120) minus WT (*n* = 120) 58.9 [95CI -213; 312]	Yes	t(238) = 0.44399, *p* = .6604	
Startle Response	Startle Latency 32dB	wh^{61}	3	111	83	Unpaired mean difference of *cyp20a1-/-* (*n* = 83) minus WT (*n* = 111) -3.34 [95CI -14.1; 8.58]	No		U = 3685, *p* = .017*
Startle Response	Startle Latency 38dB	wh^{61}	3	133	123	Unpaired mean difference of *cyp20a1-/-* (*n* = 123) minus WT (*n* = 133) 2.02 [95CI -5.13; 8.93]	No		U = 7395, *p* = .185
Startle Response	Startle Latency 41dB	wh^{61}	3	135	124	Unpaired mean difference of *cyp20a1-/-* (*n* = 124) minus WT (*n* = 135) 3.02 [95CI -1.96; 8.68]	No		U = 7805, *p* = .348
Startle Response	Startle Latency 43dB	wh^{61}	3	138	127	Unpaired mean difference of *cyp20a1-/-* (*n* = 127) minus WT (*n* = 138) 0.0176 [95CI -4.88; 5.28]	No		U = 7977, *p* = .207
Startle Response	Short Latency C-Bend Bias 32dB	wh^{61}	3	111	83	Unpaired mean difference of *cyp20a1-/-* (*n* = 83) minus WT (*n* = 111) 0.344 [95CI 0.0934; 0.582]			
Startle Response	Short Latency C-Bend Bias 38dB	wh^{61}	3	133	123	Unpaired mean difference of *cyp20a1-/-* (*n* = 123) minus WT (*n* = 133) 0.137 [95CI -0.0474; 0.34]			
Startle Response	Short Latency C-Bend Bias 41dB	wh^{61}	3	135	124	Unpaired mean difference of *cyp20a1-/-* (*n* = 124) minus WT (*n* = 135) 0.0345 [95CI -0.131; 0.212]			
Startle Response	Short Latency C-Bend Bias 43dB	wh^{61}	3	138	127	Unpaired mean difference of *cyp20a1-/-* (*n* = 127) minus WT (*n* = 138) 0.0327 [95CI -0.141; 0.201]			

Startle Response	Fraction Responding 32dB	wh^{61}	3	140	129	Unpaired mean difference of *cyp20a1-/-* (*n* = 129) minus WT (*n* = 140) -0.148 [95CI -0.24; -0.055]	No		U = 7092, *p* = .002**
Startle Response	Fraction Responding 38dB	wh^{61}	3	140	131	Unpaired mean difference of *cyp20a1-/-* (*n* = 131) minus WT (*n* = 140) -0.0154 [95CI -0.0901; 0.0587]	No		U = 8913, *p* = .637
Startle Response	Fraction Responding 41dB	wh^{61}	3	140	129	Unpaired mean difference of *cyp20a1-/-* (*n* = 129) minus WT (*n* = 140) -0.0444 [95CI -0.101; 0.00953]	No		U = 8122, *p* = .036*
Startle Response	Fraction Responding 43dB	wh^{61}	3	140	130	Unpaired mean difference of *cyp20a1-/-* (*n* = 130) minus WT (*n* = 140) -0.0536 [95CI -0.103; -0.00462]	No		U = 8002, *p* = .008**
Novel Tank Assay (Trial1)	Time In Bottom Third	wh^{61}	1	22	22	Unpaired mean difference of *cyp20a1-/-* (*n* = 22) minus WT (*n* = 22) 35.6 [95CI 25.7; 44.4]	Yes	t(42) = 7.423, *p* < .001***	
Novel Tank Assay (Trial2)	Time In Bottom Third	wh^{61}	1	22	22	Unpaired mean difference of *cyp20a1-/-* (*n* = 22) minus WT (*n* = 22) 41.3 [95CI 31.2; 50.3]	Yes	t(42) = 8.430, *p* < .001***	
Novel Tank Assay (Trial3)	Time In Bottom Third	wh^{61}	1	22	22	Unpaired mean difference of *cyp20a1-/-* (*n* = 22) minus WT (*n* = 22) 31.8 [95CI 22.3; 40.8]	Yes	t(42) = 6.646, *p* < .001***	
Novel Tank Assay (Trial1)	Latency For First Entry	wh^{61}	1	22	22	Unpaired mean difference of *cyp20a1-/-* (*n* = 22) minus WT (*n* = 22) 118 [95CI 73.1; 183]	Some		U = 54, *p* < .001***
Novel Tank Assay (Trial2)	Latency For First Entry	wh^{61}	1	22	22	Unpaired mean difference of *cyp20a1-/-* (*n* = 22) minus WT (*n* = 22) 151 [95CI 80; 225]	Some		U = 103.5, *p* < .001***
Novel Tank Assay (Trial3)	Latency For First Entry	wh^{61}	1	22	22	Unpaired mean difference of *cyp20a1-/-* (*n* = 22) minus WT (*n* = 22) 69.9 [95CI -8.18; 142]	Some		U = 176, *p* = .123
Novel Tank Assay (Trial1)	Latency For Second Entry	wh^{61}	1	22	22	Unpaired mean difference of *cyp20a1-/-* (*n* = 22) minus WT (*n* = 22) 128 [95CI 75.1; 193]	Some		U = 72.5, *p* < .001***
Novel Tank Assay (Trial2)	Latency For Second Entry	wh^{61}	1	22	22	Unpaired mean difference of *cyp20a1-/-* (*n* = 22) minus WT (*n* = 22) 76.6 [95CI -8.86; 161]	Some		U = 178, *p* = .135
Novel Tank Assay (Trial3)	Latency For Second Entry	wh^{61}	1	22	22	Unpaired mean difference of *cyp20a1-/-* (*n* = 22) minus WT (*n* = 22) 107 [95CI 9.49; 192]	Some		U = 159, *p* = .051
Novel Tank Assay (Trial1)	Total Distance Traveled	wh^{61}	1	22	22	Unpaired mean difference of *cyp20a1-/-* (*n* = 22) minus WT (*n* = 22) -52.6 [95CI -3600; 4390]	Yes	t(42) = 0.02575, *p* = .980	
Novel Tank Assay (Trial2)	Total Distance Traveled	wh^{61}	1	22	22	Unpaired mean difference of *cyp20a1-/-* (*n* = 22) minus WT (*n* = 22) -3260 [95CI -7310; 1510]	Some		U = 158, *p* = .049*
Novel Tank Assay (Trial3)	Total Distance Traveled	wh^{61}	1	22	22	Unpaired mean difference of *cyp20a1-/-* (*n* = 22) minus WT (*n* = 22) -3650 [95CI -8090; 1830]	Some		U = 147, *p* = .025*

Novel Tank Assay (Trial1)	No Transitions Top Half	wh^{61}	1	22	22	Unpaired mean difference of *cyp20a1-/-* (*n* = 22) minus WT (*n* = 22) -17.2 [95CI -30; -5.28]	Yes	t(42) = 2.595, *p* = .013*	
Novel Tank Assay (Trial2)	No Transitions Top Half	wh^{61}	1	22	22	Unpaired mean difference of *cyp20a1-/-* (*n* = 22) minus WT (*n* = 22) -26.1 [95CI -38.6; -12.9]	Some		U = 81, *p* < .001***
Novel Tank Assay (Trial3)	No Transitions Top Half	wh^{61}	1	22	22	Unpaired mean difference of *cyp20a1-/-* (*n* = 22) minus WT (*n* = 22) -26.9 [95CI -37.8; -17]	Some		U = 74.50, *p* < .001***
Novel Tank Assay (Trial1)	Total Freeze Time	wh^{61}	1	22	22	Unpaired mean difference of *cyp20a1-/-* (*n* = 22) minus WT (*n* = 22) 1.27 [95CI -5.32; 13.9]	No		U = 218.5, *p* = .588
Novel Tank Assay (Trial2)	Total Freeze Time	wh^{61}	1	22	22	Unpaired mean difference of *cyp20a1-/-* (*n* = 22) minus WT (*n* = 22) -0.182 [95CI -9.91; 24.7]	No		U = 123, *p* = .004**
Novel Tank Assay (Trial3)	Total Freeze Time	wh^{61}	1	22	22	Unpaired mean difference of *cyp20a1-/-* (*n* = 22) minus WT (*n* = 22) -1.32 [95CI -17.5; 16.2]	No		U = 197, *p* = .297
Novel Tank Assay (Trial1)	No Freezing Episodes	wh^{61}	1	22	22	Unpaired mean difference of *cyp20a1-/-* (*n* = 22) minus WT (*n* = 22) 1.36 [95CI -4.64; 9.41]	No		U = 241.5, *p* = .995
Novel Tank Assay (Trial2)	No Freezing Episodes	wh^{61}	1	22	22	Unpaired mean difference of *cyp20a1-/-* (*n* = 22) minus WT (*n* = 22) -4.23 [95CI -12.1; 6.06]	No		U = 155.5, *p* = .042*
Novel Tank Assay (Trial3)	No Freezing Episodes	wh^{61}	1	22	22	Unpaired mean difference of *cyp20a1-/-* (*n* = 22) minus WT (*n* = 22) -4.45 [95CI -13.8; 3.23]	Some		U = 217, *p* = .564
Novel Tank Assay (Trial1)	No Darting Episodes	wh^{61}	1	22	22	Unpaired mean difference of *cyp20a1-/-* (*n* = 22) minus WT (*n* = 22) -7.68 [95CI -14.8; -1.93]	Yes	t(42) = 2.294, *p* = .027*	
Novel Tank Assay (Trial2)	No Darting Episodes	wh^{61}	1	22	22	Unpaired mean difference of *cyp20a1-/-* (*n* = 22) minus WT (*n* = 22) -6.68 [95CI -13.1; 0]	Yes	t(42) = 1.930, *p* = .060	
Novel Tank Assay (Trial3)	No Darting Episodes	wh^{61}	1	22	22	Unpaired mean difference of *cyp20a1-/-* (*n* = 22) minus WT (*n* = 22) -5.82 [95CI -12.8; 0.853]	Yes	t(42) = 1.643, *p* = .108	

[1] t(degrees of freedom) = the t statistic, *p* = p-value

[2] U = the U statistic, *p* = p value

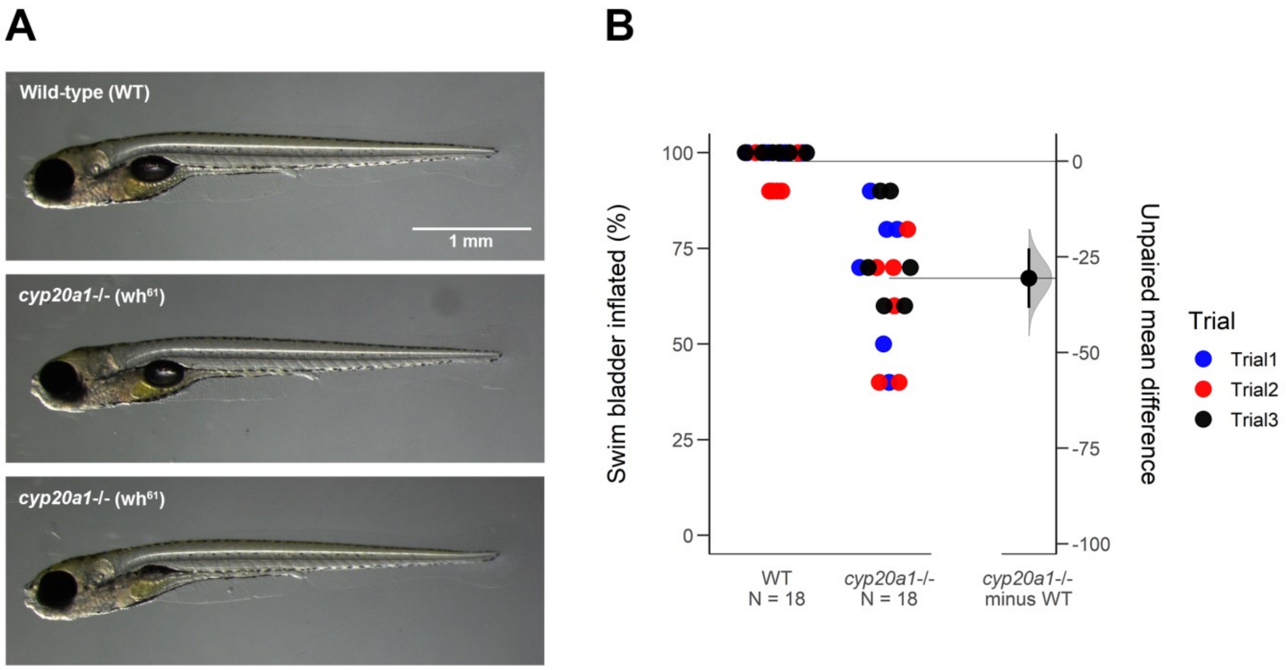

Figure S1. Morphology of *cyp20a1-/-* larvae at 6 dpf. (**A**) *cyp20a1-/-* (wh^{61}) mutant larvae show no apparent morphological differences in comparison to the wild-type (WT) line except for (**B**) swim bladder inflation, which was reduced in the mutant larvae. The experiment was repeated three times independently with 6 dishes containing 10 larvae per experiment (total *n* of 3 experiments = 18).

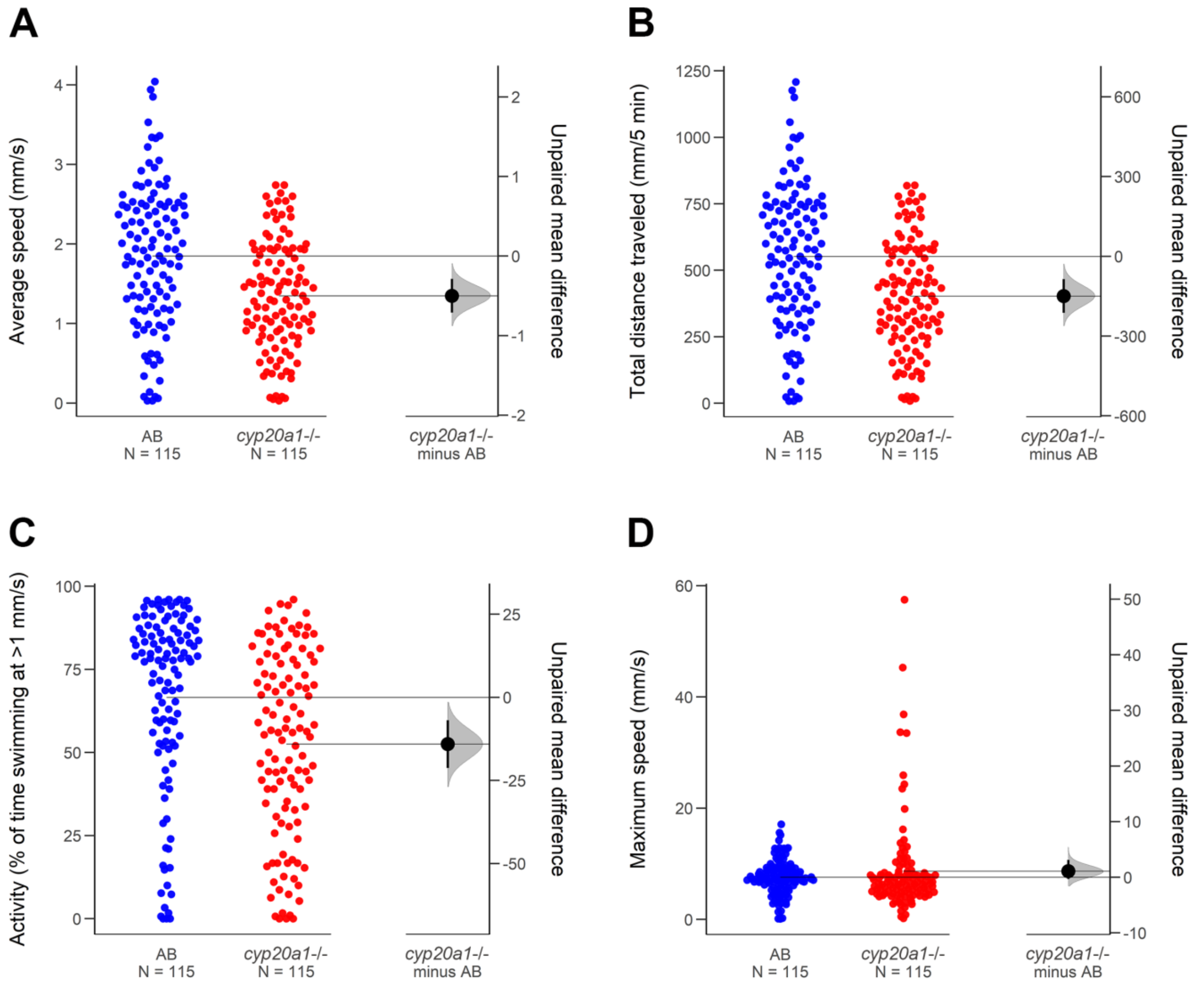

Figure S2. Activity of larvae prior to optomotor response (OMR) assay. (**A**) Average speed, (**B**) total distance traveled, (**C**) activity, and (**D**) maximum speed was measured in the 5 minutes prior to the OMR assay. A total of 120 larvae per fish line (WT and *cyp20a1-/-* wh[61]) were recorded.

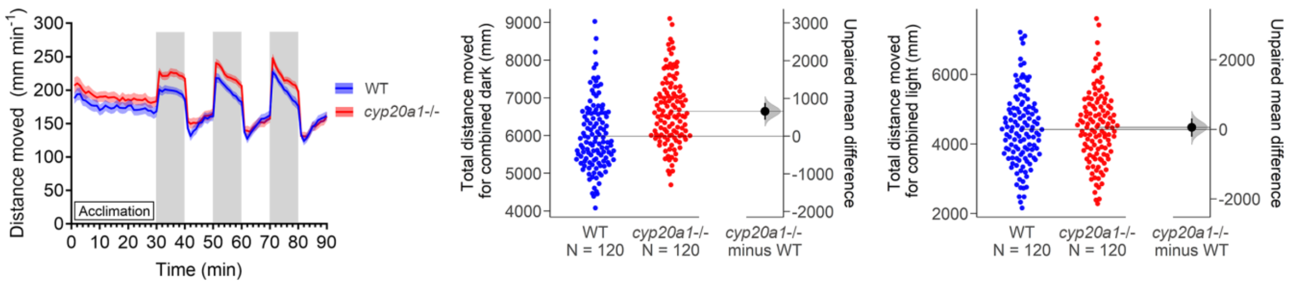

Figure S3. Locomotion during the light-dark assay of *cyp20a1-/-* (wh[60]) mutant larvae in comparison to WT. *cyp20a1-/-* (wh[60]) mutant larvae show hyperactivity in the dark phase but not in the light phase. The experiment was repeated five times independently (total *n* = 120).

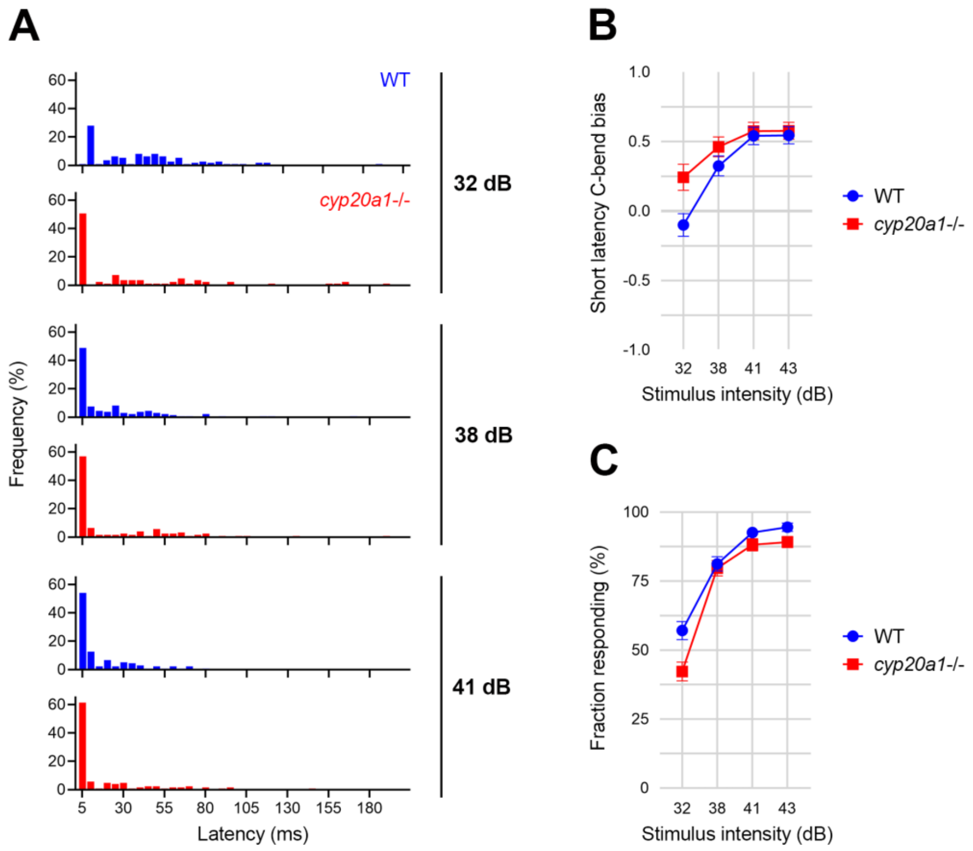

Figure S4. Startle response. (**A**) Latency of WT and *cyp20a1-/-* (wh^{61}) mutant larvae at 32 dB, 38 dB, and 41 dB. For 43 dB see Figure 2. (**B**) Bias toward short-latency C-bend (< 15 ms) at all stimulus intensities. (**C**) Fraction of larvae responding at different stimulus intensities. All data from three independent experiments.

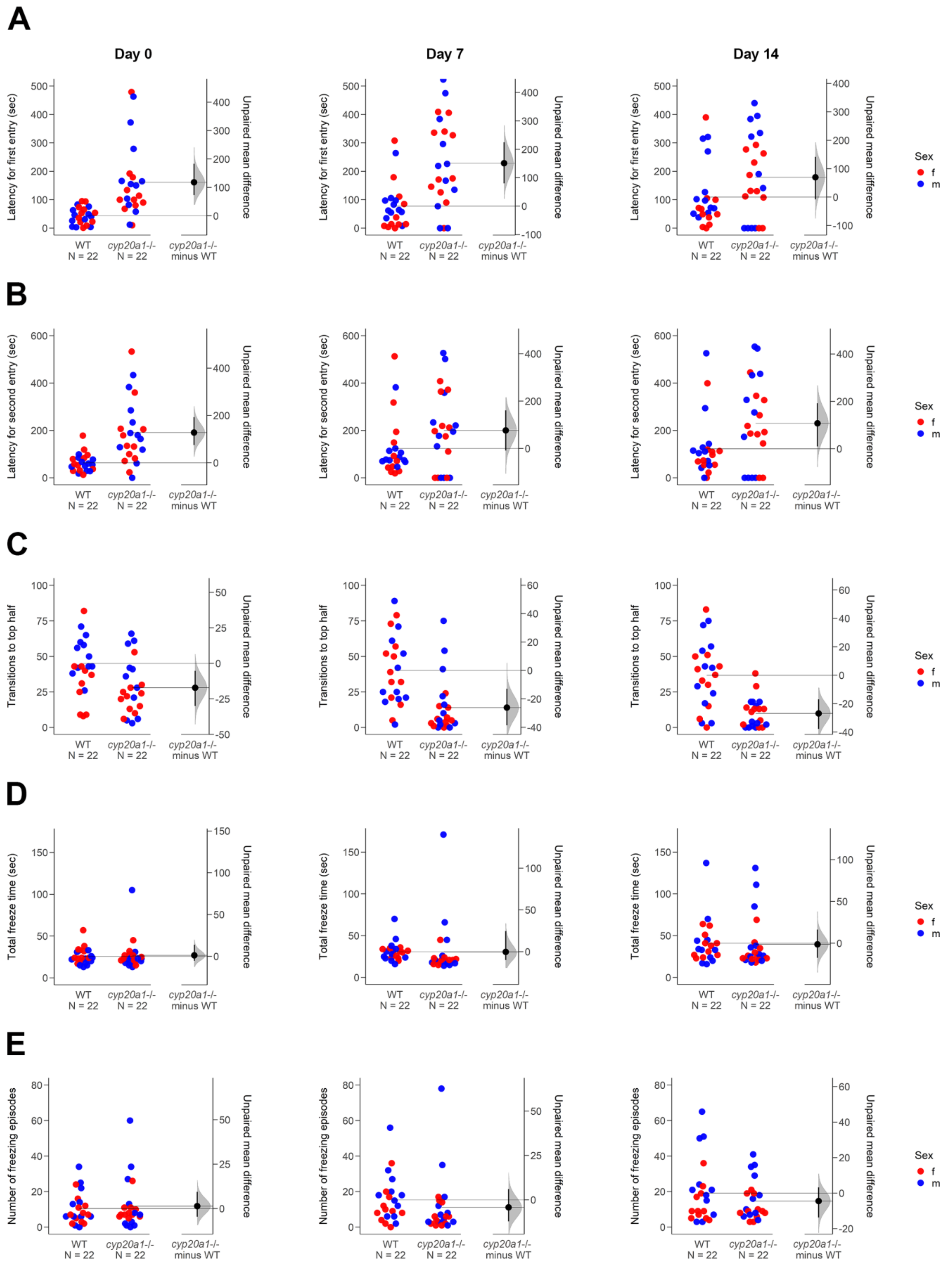
A
B
C
D
E
Day 0
Day 7
Day 14
Latency for first entry (sec)
Latency for second entry (sec)
Transitions to top half
Total freeze time (sec)
Number of freezing episodes
Unpaired mean difference
WT
N = 22
cyp20a1-/-
N = 22
cyp20a1-/-
minus WT
Sex
f
m

F

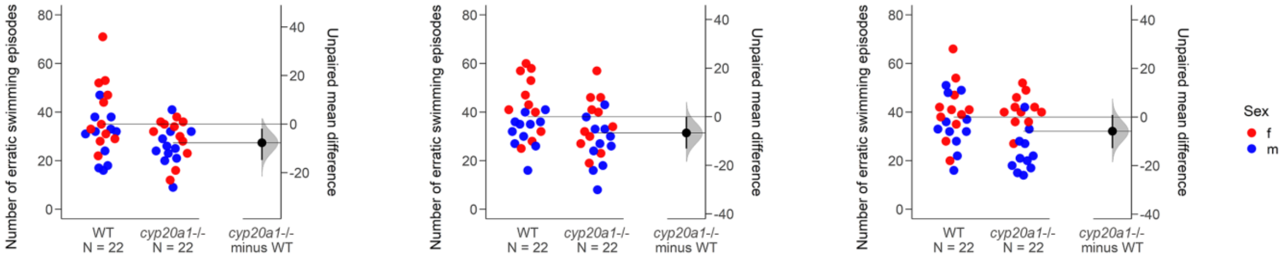

Figure S5. Adult WT and *cyp20a1-/-* (wh[61]) behavior in the novel tank assay. (**A**) Latency for first entry to the top half, (**B**) latency for second entry to the top half, (**C**) number of transitions to the top half, (**D**) total freeze time, (**E**) number of freezing episodes, (**F**) number of erratic swimming episodes (also called darting). The experiment was repeated on day 7 and day 14 with the same fish.
