# Supplementary material for: Orphan cytochrome P450 20a1 CRISPR/Cas9 mutants and neurobehavioral phenotypes in zebrafish
Source: Sci Rep. 2021 Dec 13;11:23892. doi: 10.1038/s41598-021-03068-3 (PMC8669017; doi:10.1038/s41598-021-03068-3)
Supplement: Supplementary file 2 — Supplementary Legends. [file 41598_2021_3068_MOESM2_ESM.docx]

The source data underlying the findings of this study are available within the Supplementary Data 1.
